# Effect of Dexmedetomidine on Postoperative Analgesia After Video-assisted Thoracoscopic Lobectomy Surgery

**DOI:** 10.7150/ijms.136546

**Published:** 2026-07-30

**Authors:** Dowon Lee, Eun Ji Park, Jae-young Kwon, Hyae Jin Kim, Siyun Moon, Boo-young Hwang

**Affiliations:** 1 Department of Anesthesia and Pain Medicine, Biomedical Research Institute, Pusan National University Hospital, Busan, South Korea.; 2 Department of Anesthesia and Pain Medicine, School of Medicine, Pusan National University, Yangsan, South Korea.

**Keywords:** adrenergic alpha-2 receptor agonists, analgesia, dexmedetomidine, patient-controlled analgesia, postoperative pain, video assisted thoracoscopic surgery

## Abstract

**Background:**

Dexmedetomidine is a highly selective α2-adrenergic receptor agonist that provides sedative and analgesic effects and may reduce opioid requirements without significant respiratory depression. However, evidence supporting its use in patients undergoing video-assisted thoracoscopic (VATS) lobectomy remains limited. This study aimed to evaluate the effects of perioperative dexmedetomidine on postoperative analgesic requirements and recovery outcomes in patients receiving combined epidural and general anesthesia.

**Materials and Methods:**

This prospective, randomized, double-blind study enrolled 68 patients undergoing elective VATS lobectomy. Patients were randomly assigned to the dexmedetomidine group (Group D) or the saline control group (Group S). All patients received standardized general anesthesia combined with thoracic epidural blockade. Group D received an intravenous loading dose of dexmedetomidine (1.0 μg/kg over 10 minutes), followed by a continuous infusion at 0.5 μg/kg/h until 1 hour after surgery, whereas Group S received a volume-matched saline infusion. The primary outcome was total patient-controlled epidural analgesia (PCEA) consumption. Secondary outcomes included visual analog scale (VAS) scores, Ramsay sedation scores, adverse events, and patient satisfaction assessed over 48 hours postoperatively.

**Results:**

Sixty-one patients were included in the final analysis (Group D, n = 31; Group S, n = 30). Baseline demographic and intraoperative characteristics were comparable between groups. Intraoperative rocuronium requirements, cumulative patient-controlled epidural analgesia (PCEA) consumption from the intraoperative period to 48 hours postoperatively, and the number of rescue analgesic boluses administered in the post-anesthesia care unit were significantly lower in Group D than in Group S (p = 0.002, 0.001, and 0.013, respectively). Although overall postoperative pain scores were comparable between groups, VAS scores at 1 hour postoperatively were significantly lower in Group D (p = 0.038). Patient satisfaction scores were significantly higher in Group D (p = 0.005). Bradycardia 1 hour after surgery and hypotension 1 hour after one-lung ventilation occurred more frequently in Group D (p = 0.044 and 0.024, respectively), whereas the incidence of other adverse events was similar between groups.

**Conclusions:**

Perioperative dexmedetomidine infusion reduced postoperative epidural analgesic requirements and improved early postoperative pain control and patient satisfaction in patients undergoing VATS lobectomy with combined epidural and general anesthesia. Despite an increased incidence of transient bradycardia and hypotension, dexmedetomidine was well tolerated and may be considered an effective opioid-sparing adjunct in this surgical population

## Introduction

Thoracic surgery, including video-assisted thoracoscopic surgery (VATS), is associated with substantial postoperative pain that can impair respiratory function, delay mobilization, and increase the risk of pulmonary complications and prolonged hospitalization 1. Although thoracic epidural analgesia remains a standard postoperative analgesic strategy for thoracic surgery, many patients still require supplemental opioid administration for breakthrough pain, which may contribute to opioid-related adverse effects such as postoperative nausea and vomiting, excessive sedation, and respiratory depression 2,3. Consequently, multimodal opioid-sparing analgesic approaches have become increasingly important in enhanced recovery after surgery pathways for thoracic procedures 4.

Dexmedetomidine, a highly selective α2-adrenergic receptor agonist, possesses sedative, anxiolytic, sympatholytic, and analgesic properties with minimal respiratory depression 5. Previous studies have demonstrated that perioperative dexmedetomidine administration can reduce anesthetic and opioid requirements while improving postoperative analgesia and recovery quality 6-8. However, evidence regarding its perioperative use in patients undergoing VATS lobectomy with concomitant thoracic epidural analgesia remains limited.

Therefore, this prospective randomized study aimed to evaluate the effects of perioperative dexmedetomidine infusion on postoperative analgesic consumption, pain intensity, and recovery profiles in patients undergoing VATS lobectomy. We hypothesized that perioperative dexmedetomidine administration would reduce postoperative opioid requirements and improve postoperative pain control without increasing adverse events.

## Materials and Methods

### Ethical Statement

This prospective randomized study was conducted between January and October 2024 in the Department of Anesthesia and Pain Medicine at Pusan National University Hospital, Korea. The study was approved by the Institutional Review Board (IRB) of Pusan National University Hospital (No. 2502-019-148) and prospectively registered at the Clinical Research Information Service (CRIS, KCT0010962), a primary registry of the World Health Organization International Clinical Trials Registry Platform. All patients provided written informed consent prior to surgery.

### Participants

A total of 68 patients aged 40-75 years with American Society of Anesthesiologists (ASA) physical status I-III who were scheduled for elective VATS lobectomy were assessed for eligibility. Patients who were contraindicated for treatment with local anesthetics, opioids, nonsteroidal anti-inflammatory drugs, or epidural analgesia and who had psychological disorder, chronic pain disorder, or preoperative administration of drugs, including opioids, antidepressants, gabapentin, pregabalin, and carbamazepine, were excluded. Patients who could not use the visual analog scale (VAS); had increased intracranial pressure, renal failure, or hepatic failure; or were pregnant were also excluded. Finally, 64 patients were enrolled in this study. Patients excluded because of epidural anesthesia failure were removed prior to final outcome analysis. No missing postoperative outcome data were observed among the analyzed participants.

### Intervention

The patients were divided into two groups: Group D (dexmedetomidine) and Group S (saline). Patients were randomly assigned in a 1:1 ratio to either the Group D or Group S using a computer-generated randomization sequence. Group allocation was concealed in sequentially numbered, opaque, sealed envelopes that were opened immediately before anesthesia induction by an investigator who was not involved in patient management or outcome assessment. Simple randomization without blocking or stratification was used. The study drugs were prepared in identical syringes by an investigator not involved in anesthetic management, postoperative assessment, or data analysis. Dexmedetomidine and normal saline were administered in equal volumes using the same infusion protocol to maintain blinding. Patients, anesthesiologists, postoperative outcome assessors, and data analysts were blinded to group allocation throughout the study period. Anesthesia was standardized. Glycopyrrolate 0.2 mg was intramuscularly injected into all patients 30 minutes before induction of anesthesia. After the patient entered the operating room, basic vital signs such as baseline heart rate (HR), systolic blood pressure (SBP), diastolic blood pressure (DBP), mean arterial blood pressure (MBP), pulse oximetry oxygen saturation (SpO_2_), respiratory rate (RR), and central venous pressure (CVP) were checked, the patient was placed in the right lateral position, and an epidural injection was performed using the paramedian method at the level of the fourth thoracic vertebra. Bispectral index (BIS, XP version 4.1; Aspect Medical Systems, Newton, MA, USA) monitoring was used to measure the depth of anesthesia. After confirming the stability of the procedure by administering a test dose (1.5% lidocaine 3 ml with 1:200,000 epinephrine), a loading dose of 0.125% Naropin 4 ml was injected. Propofol 2 mg/kg i.v. and rocuronium 0.6 mg/kg i.v. were used for the induction, and desflurane was used for maintenance. The cocktail drug for epidural anesthesia was made by mixing 40 ml of 0.75% ropivacaine hydrochloride and 500 mcg of fentanyl in 150 ml of normal saline. Rocuronium 10 mg was administered when Train-of-Four (TOF) was over 2/4. An arterial catheter was maintained before oxygen inhalation, and arterial blood gas analysis (ABGA) results were obtained using an epoc Host blood analysis system (Alere, USA). During one-lung ventilation (OLV), a lung-protective ventilation strategy was applied using a tidal volume of 5-6 mL/kg predicted body weight, positive end-expiratory pressure (PEEP) of 5 cmH₂O, and adjustment of inspired oxygen fraction (FiO₂) to maintain SpO_2_ > 92%. Patients in Group D received an intravenous loading dose of dexmedetomidine (1.0 μg/kg) administered over 10 minutes, followed by a continuous infusion at 0.5 μg/kg/h, whereas Group S received volumetrically matched normal saline based on prior study^9^. The infusion was maintained until the end of surgery and continued for an additional hour after admission to the post-anesthesia care unit (PACU).

Bradycardia, defined as a HR < 45 beats/min, was treated with intravenous atropine sulfate hydrate 0.5 mg. Hypotension, defined as SBP < 90 mmHg or a decrease > 20% from baseline, was managed with intravenous ephedrine (5 mg boluses) and additional crystalloid fluid administration as clinically indicated. An antiemetic (ondansetron 8 mg i.v.) was administered 30 minutes before the end of surgery. Postoperatively, pyridostigmine 10 mg i.v. and glycopyrrolate 0.4 mg i.v. were administered. Patients were transferred to the PACU and stayed there until their Aldrete score was greater than 8. Patients received pain control via a patient-controlled analgesia (PCA) device (GemStar® Infusion System, Hospira, IL, USA) with a bolus dose of 3 ml and a lock-out interval of 20 minutes. Patients were instructed to press the button whenever the VAS was 4 or higher.

### Assessment

The primary outcome was cumulative postoperative PCA consumption during the first 48 hours after surgery. We measured vital signs and performed ABGA seven times (before surgery; 30 and 60 minutes after one-lung ventilation [OLV]; and 1, 6, 24, and 48 hours after surgery). After the operation, a blinded observer assessed the infused PCA dose, pain using the VAS at rest, and the Richmond Agitation-Sedation Scale (RASS) sedation scale, which ranges from -5 (unarousable) to +4 (combative), with 0 indicating an alert and calm state at 1, 6, 24, and 48 hours. Side effects such as nausea, vomiting, headache, shivering, and pruritus were assessed at 1, 6, 24, and 48 hours and satisfaction at 48 hours after the operation. Nausea was classified into three grades: 1 = mild, 2 = moderate, and 3 = severe. If patients complained of nausea above grade 2, ondansetron 4 mg i.v. was administered. Vomiting was classified into two grades: 1 = < 4 times of vomiting; 2 = ≥ 4 times of vomiting. Patients were asked to rank their satisfaction according to the following scale: 1 = very unsatisfactory, 2 = unsatisfactory, 3 = neutral, 4 = satisfactory, and 5 = very satisfactory.

### Statistical Analysis

Sample size was predetermined using the z-test in G power, and selecting the following parameters: tails = two, α error prob = 0.05, power (1-β error prob) = 0.8, and allocation ratio N2/N1 = 1. Sample size calculation was performed based on the primary outcome of cumulative postoperative PCA consumption during the first 48 hours after surgery. Because previous perioperative analgesic studies commonly reported postoperative opioid consumption at 24 hours, effect size estimation was based on prior 24-hour opioid consumption data. A medium effect size, corresponding to an approximately 30% reduction in postoperative opioid requirement based on previous literature, was considered clinically meaningful 10. Accordingly, the sample size was calculated to be 31 patients in each group. Although cumulative PCA consumption was prospectively assessed over 48 postoperative hours in the present study, the observed reduction at 24 hours was consistent with the anticipated effect size used for sample size estimation. Considering the dropout rate of 10%, 34 patients were assigned to each group. Data are expressed as mean ± standard deviation. Continuous variables are presented as mean ± standard deviation and were compared using Student's t-test. Categorical variables are presented as number (percentage) and were analyzed using the chi-square test. The incidence of side effects was compared between the two groups using the chi-square test and Mann-Whitney U test. Vital signs, cumulative PCA dose, and VAS were compared using two-way repeated measures analysis of variance (two-way RM-ANOVA) and the Mann-Whitney U test. The satisfaction scores of the two groups were compared using the Mann-Whitney U test. Statistical significance was set at p < 0.05. SPSS (version 21.0, IBM Statistics Data Editor; IBM Corp., Armonk, NY, USA) was used for all statistical analyses. Patients excluded because of epidural anesthesia failure were removed prior to final outcome analysis. No missing postoperative outcome data were observed among the analyzed participants; therefore, imputation methods were not required.

## Results

**Figure 1:**
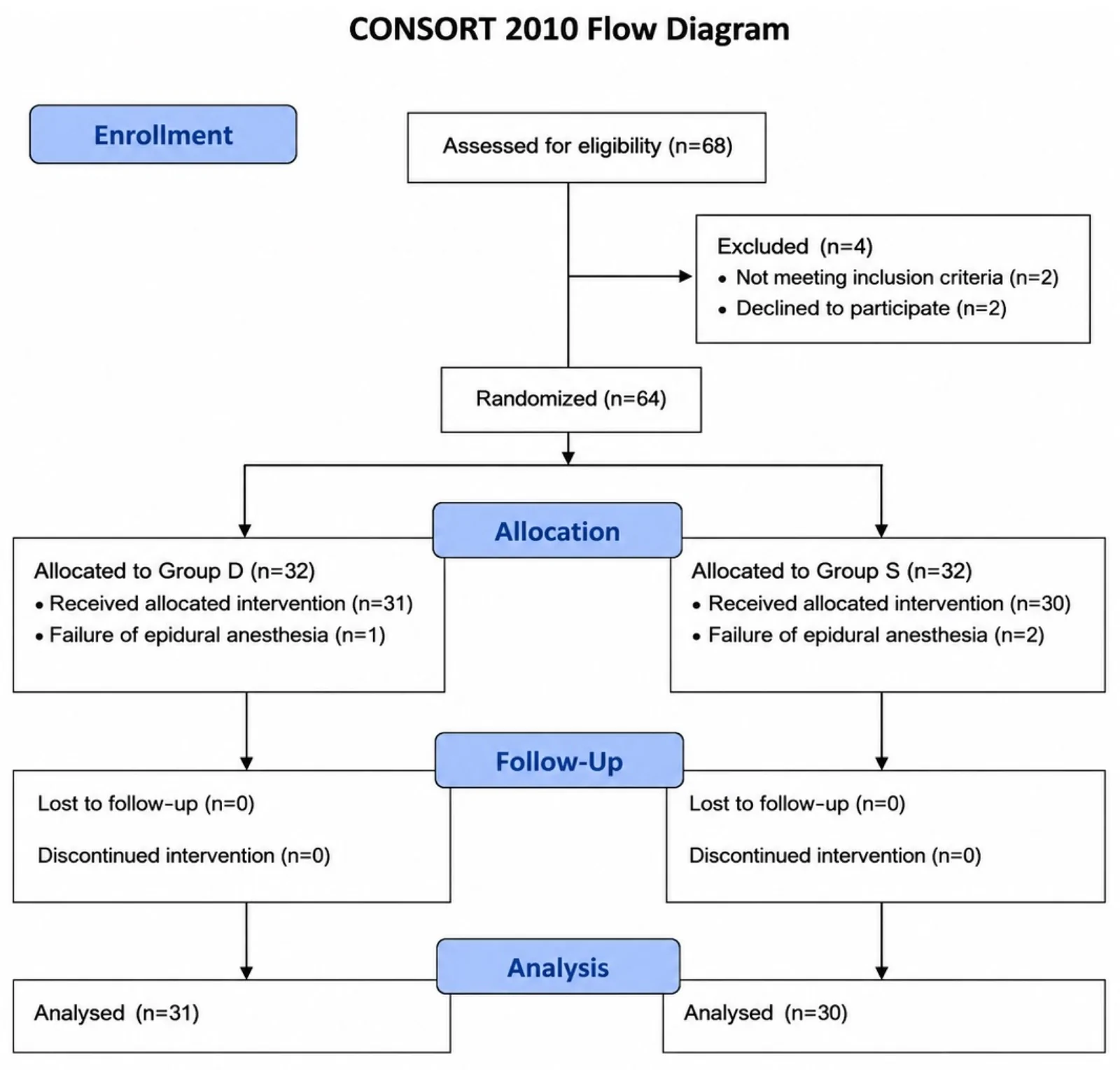
CONSORT flow diagram of patient enrollment and analysis. A total of 68 patients were assessed for eligibility, of whom 4 were excluded due to operation cancellation. The remaining 64 patients were randomized into Group D (n = 32) and Group S (n = 32). One patient in Group D and two in Group S were excluded due to failure of epidural anesthesia. Ultimately, 61 patients (Group D, n = 31; Group S, n = 30) were included in the final analysis.

A total of 68 patients were enrolled in the study, and 61 were ultimately included in the final analysis. Four patients cancelled the operation, and 64 patients were randomized into Groups D and S. One patient in Group D and two in Group S were excluded from the trial because of failure of epidural anesthesia (Figure 1).

Basic information included patient sex, height, weight, underlying conditions such as hypertension and diabetes mellitus, smoking history, and ASA physical status. There were no significant differences between the groups (Table 1).

Anesthesia time, operation time, and administered ephedrine count did not differ between the two groups. The administered rocuronium count, administered bolus count of analgesics in the PACU, and administered PCA dose during surgery were lower in Group D than in Group S (p = 0.002, 0.013, and 0.001, respectively, Table 2). No significant differences were observed between groups with respect to OLV duration or intraoperative ventilatory parameters, including tidal volume, PEEP, and FiO₂ (p = 0.333, 0.292, 0.311). The amount of fluid administered was higher in Group D than in Group S (p = 0.03, Table 2). The RASS in the PACU differed between the two groups, and the satisfaction score of Group D was higher than that of Group S (p = 0.046, 0.005, Table 2).

**Figure 2:**
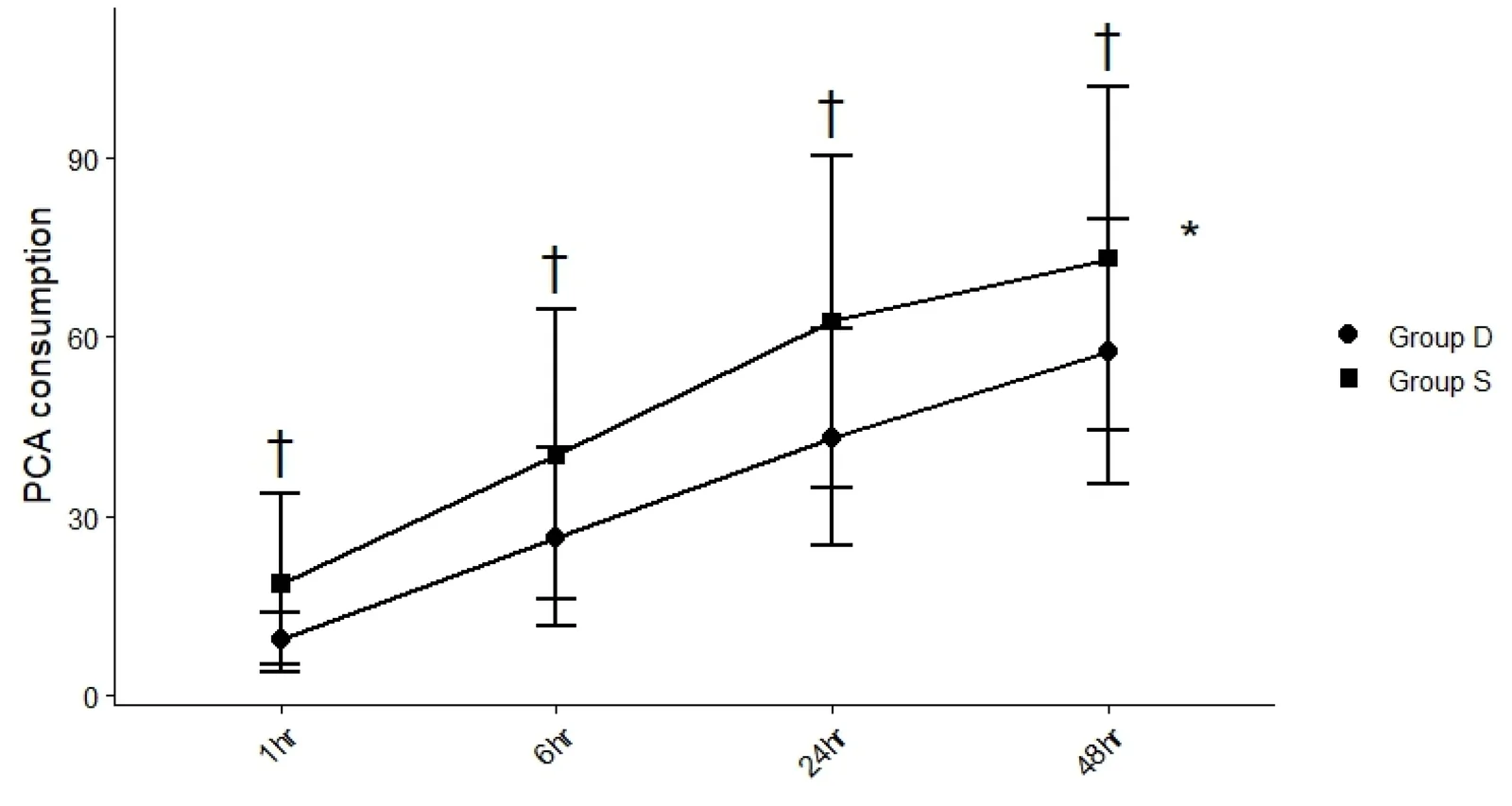
Cumulative patient-controlled analgesia (PCA) consumption over time. Cumulative PCA analgesic consumption was significantly lower in Group D compared to Group S throughout the study period (p = 0.001). Data are presented as mean ± standard deviation. *P < 0.05 for overall group difference by repeated-measures analysis of variance (RM-ANOVA); †P < 0.05 between groups at the corresponding time point.

**Figure 3:**
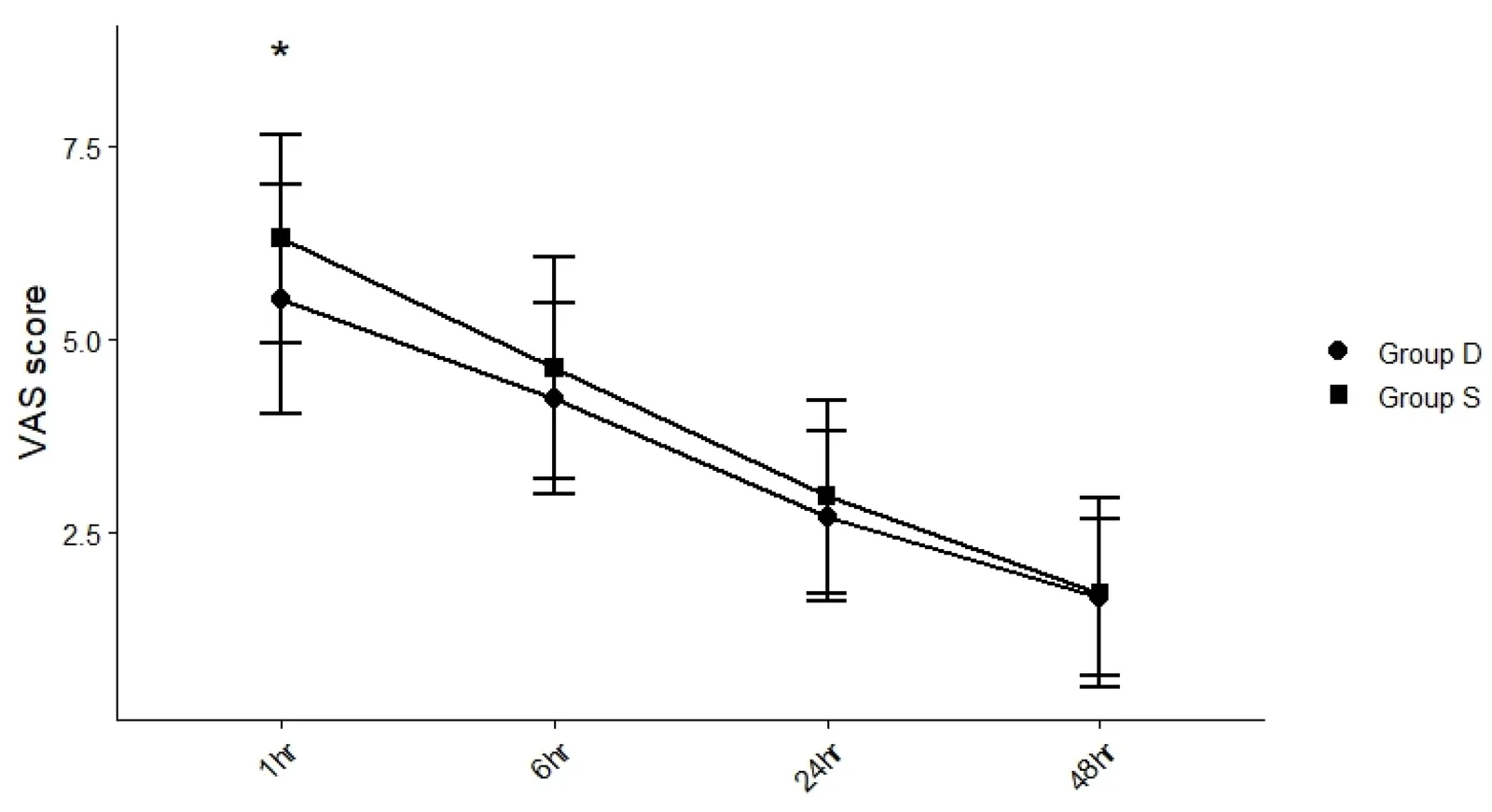
Postoperative pain intensity measured by visual analog scale (VAS). VAS scores for pain intensity did not differ significantly between groups over the study period. However, VAS scores were significantly lower in Group D at 1 hour postoperatively. Data are presented as mean ± standard deviation. *P < 0.05 between groups at the corresponding time point.

Cumulative PCA consumption was significantly lower in Group D than in Group S over the entire postoperative period (RM-ANOVA, p = 0.001, Figure 2). At individual postoperative time points, cumulative PCA consumption was significantly lower in Group D than in Group S at 1 hour (9.58 ± 4.20 vs. 18.8 ± 14.97 mL, p = 0.004), 6 hours (26.52 ± 14.90 vs. 40.3 ± 24.27 mL, p = 0.007), 24 hours (43.16 ± 18.08 vs. 62.53 ± 27.87 mL, p = 0.011), and 48 hours postoperatively (57.61 ± 22.07 vs. 73.1 ± 28.64 mL, p = 0.037). There was no significant overall difference in postoperative VAS scores between groups over the study period based on RM-ANOVA. However, VAS scores at 1 hour postoperatively were significantly lower in Group D than in Group S (5.52 ± 1.48 vs. 6.3 ± 1.34, p = 0.038, Figure 3).

**Figure 4:**
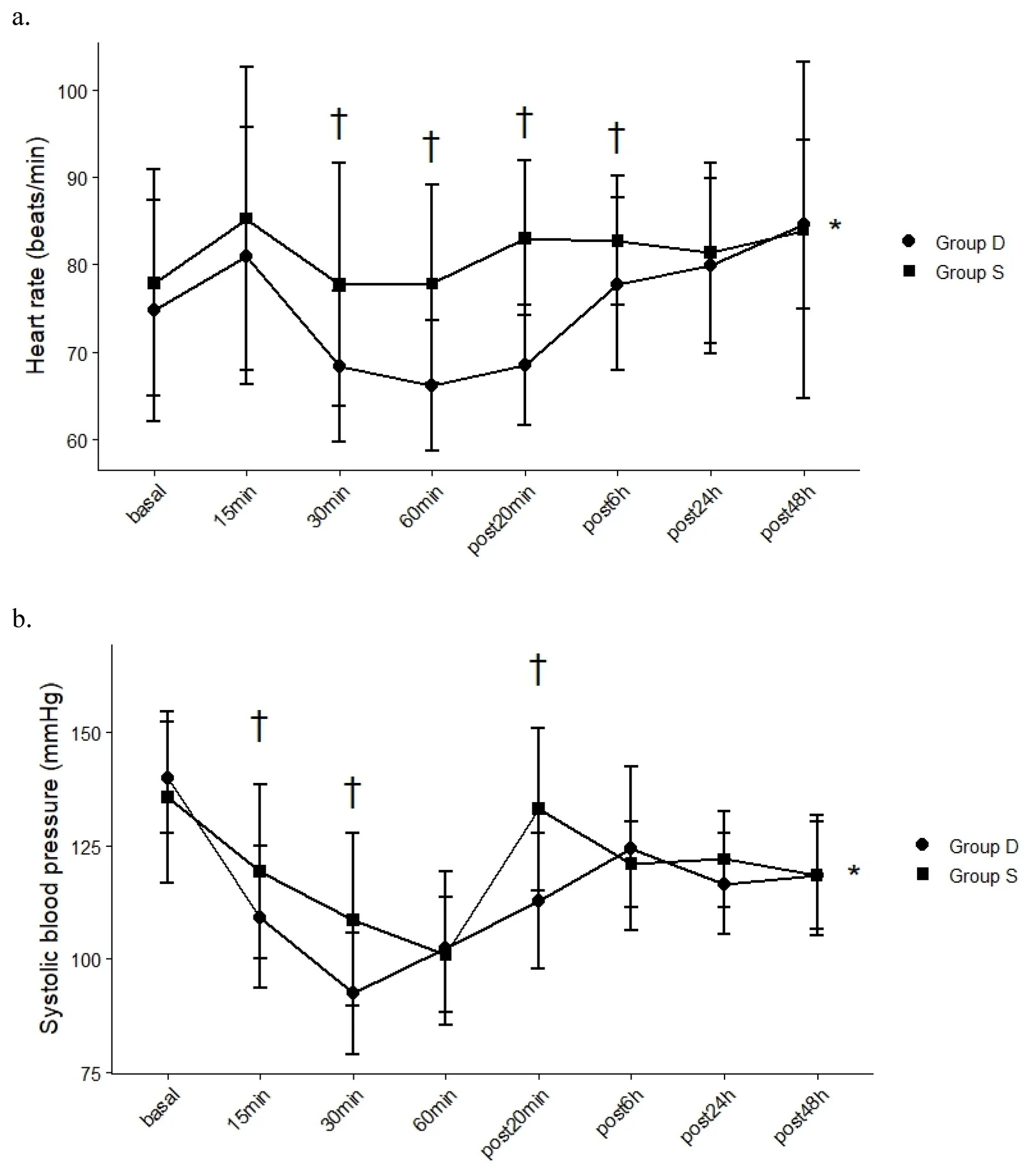

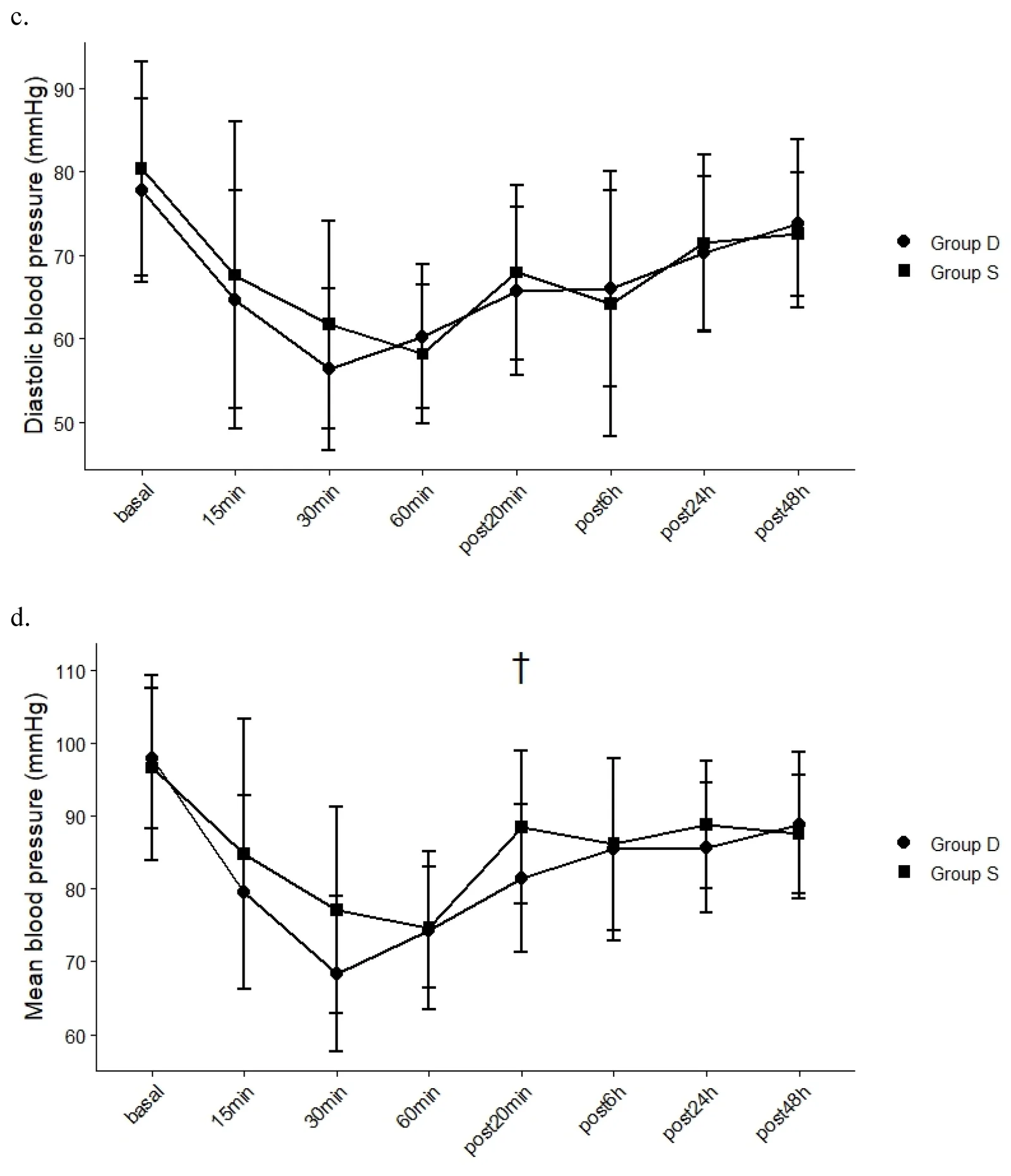
Changes in perioperative vital signs over time. (a) Heart rate (HR), (b) systolic blood pressure (SBP), (c) diastolic blood pressure (DBP), and (d) mean blood pressure (MBP) measured at each time point. HR and SBP were significantly lower in Group D compared to Group S over the study period. MBP showed temporal fluctuations without significant intergroup differences. DBP showed no significant intergroup differences. Data are presented as mean ± standard deviation. *P < 0.05 for overall group difference by repeated-measures analysis of variance (RM-ANOVA); †P < 0.05 between groups at the corresponding time point.

The incidence of adverse events such as nausea, vomiting, shivering, and respiratory depression did not differ significantly between the groups. Patient satisfaction scores were higher in Group D than in Group S (p = 0.005, Table 2). The frequencies of bradycardia 1 hour after surgery and hypotension 1 hour after OLV were higher in Group D (p = 0.044 and 0.024, respectively, Table 3). HR and SBP were lower in Group D than in Group S (p = 0.001 and 0.034, respectively, Figure 4). MBP showed temporal fluctuations, and other vital parameters, including DBP, pulse oximetry oxygen saturation (SpO₂), RR, and CVP showed no significant intergroup differences.

In serial ABGA performed seven times within 48 hours, pH values were significantly lower and the partial pressure of arterial carbon dioxide (PaCO₂) was higher in Group D than in Group S (p = 0.045, 0.042). However, PaO₂, HCO₃⁻, lactate, and SaO₂ did not differ significantly between the groups.

## Discussion

Dexmedetomidine leads to less postoperative pain, reduced opioid consumption, and a lower risk of opioid-related adverse events 11. Because excessive perioperative opioid exposure may promote opioid-induced hyperalgesia and increase postoperative opioid requirements, opioid-sparing analgesic strategies are clinically important 12. In the present study, perioperative dexmedetomidine administration significantly reduced postoperative analgesic requirements and early pain intensity following VATS lobectomy.

The observed reduction in cumulative PCA consumption over 48 hours suggests that the analgesic benefits of dexmedetomidine extend beyond the immediate postoperative period. To enhance early recovery, dexmedetomidine infusion was continued into the early postoperative phase to facilitate a smooth emergence and provide immediate opioid-sparing effects. Consistent with this strategy, the number of analgesic boluses administered in the post-anesthesia care unit was significantly lower in the dexmedetomidine group, indicating improved early postoperative comfort. Importantly, these analgesic benefits were achieved without an increase in adverse events, and patient satisfaction was higher in the dexmedetomidine group. These findings support the role of dexmedetomidine in improving the quality of perioperative pain control and recovery.

Previous studies have shown that perioperative dexmedetomidine infusion can improve postoperative recovery quality and analgesia without increasing major adverse events 6, 13, 14. Recent cohort studies have also suggested that dexmedetomidine may influence recovery characteristics in the post-anesthesia care unit, supporting the rationale for extending its use into the early recovery period.

An additional finding of this study was the significant reduction in intraoperative neuromuscular blocking agent requirements in the dexmedetomidine group. This observation is consistent with the anesthetic-sparing and sympatholytic properties of dexmedetomidine mediated through central α2-adrenergic receptor activation. α2-adrenergic agonists enhance anesthetic depth and reduce anesthetic requirements through central sympatholytic and sedative effects 15. Deeper levels of anesthesia are known to reduce the requirement for neuromuscular blocking agents 16,17. Therefore, we do not suggest a direct neuromuscular blocking effect of dexmedetomidine; rather, the reduced requirement for neuromuscular blockade is likely a secondary consequence of its well-established anesthetic-sparing effects 17,18. In the present study, this effect may have been further augmented by the concomitant use of thoracic epidural anesthesia.

Hemodynamic analysis demonstrated a significantly lower HR and SBP in the dexmedetomidine group throughout the perioperative period, which was consistent with its sympatholytic effects 19. Although these changes were statistically significant, they were not associated with clinically relevant adverse outcomes, suggesting that dexmedetomidine can be safely administered with appropriate monitoring. Other vital parameters, including DBP, SpO_2_, RR, and CVP, showed temporal changes but did not differ between the groups.

Serial arterial blood gas analyses demonstrated slightly lower pH in the dexmedetomidine group, which may be attributed to several factors. Because intraoperative ventilatory parameters and OLV duration were comparable between groups, ventilatory management is unlikely to explain the differences in pH and PaCO₂. However, after the surgery, the sedative effects of dexmedetomidine, particularly when combined with other anesthetic agents, may result in mild hypoventilation during the early recovery period, although it preserves respiratory function 20. PaCO₂ was slightly higher in Group D, suggesting mild hypoventilation during early recovery. In addition, the sympatholytic effects of dexmedetomidine may contribute to transient changes in tissue perfusion. Group D received significantly greater amounts of intravenous fluid during surgery, likely reflecting compensatory management for the sympatholytic and vasodilatory effects of dexmedetomidine. Differences in perioperative fluid administration may have influenced hemodynamic variables and respiratory parameters, including PaCO₂, and therefore should be considered as a potential confounding factor when interpreting these findings. However, because lactate levels did not differ between the groups, these findings were unlikely to represent clinically significant metabolic derangements. Although statistically significant, the magnitude of difference was modest and may not represent clinically meaningful changes.

The growing emphasis on multimodal analgesic strategies in contemporary perioperative care aligns with our findings regarding the opioid-sparing potential of dexmedetomidine. Increasing awareness of opioid-related complications has driven the interest in alternative analgesic adjuncts, and dexmedetomidine may represent a valuable component of such strategies. The integration of dexmedetomidine into thoracic epidural-based multimodal analgesic protocols may provide synergistic opioid-sparing effects without compromising safety, which may support enhanced recovery after thoracic surgery 7,21. Recent studies evaluating dexmedetomidine as an adjunct to regional anesthetic techniques have consistently demonstrated reductions in opioid consumption and prolonged analgesia 22. Our findings extend this evidence by focusing on patients undergoing VATS lobectomy with concomitant epidural analgesia. Although the observed differences were modest, they were statistically significant. Therefore, they may translate into meaningful improvements in patient comfort and recovery without increasing adverse events.

Several limitations regarding the interpretation of the results warrant acknowledgment. First, although this study was designed as a randomized, double-blind trial, the sample size was relatively small, which may limit the generalizability of the findings and reduce the statistical power to detect differences in some secondary outcomes, such as patient satisfaction and recovery indices. In addition, the sample size calculation was based on the primary outcome of postoperative analgesic consumption and may therefore have been insufficient to detect differences in several secondary endpoints, including certain hemodynamic and recovery variables. Accordingly, secondary outcome findings should be interpreted with caution and considered exploratory in nature. In addition, because multiple secondary outcomes and repeated time-point comparisons were analyzed, the possibility of type I error cannot be completely excluded. Therefore, secondary findings, particularly those with modest effect sizes, should be interpreted cautiously, as statistically significant differences may not necessarily indicate clinically meaningful effects. In addition, several patients were excluded because of epidural anesthesia failure. Although the number of exclusions was small and relatively balanced between groups, exclusion after randomization may have introduced potential selection bias. Second, although neuromuscular blockade was managed using quantitative TOF monitoring with predefined criteria for additional rocuronium administration, this study was not specifically designed to investigate the mechanistic interactions between dexmedetomidine and neuromuscular blocking agents. Therefore, while a reduction in rocuronium requirement was observed, the underlying mechanisms, such as anesthetic-sparing effects and central modulation, could not be fully elucidated. Third, dexmedetomidine infusion was continued for 1 hour into the PACU, which may have influenced early postoperative respiratory parameters, including PaCO₂ and arterial pH. Although these changes were mild and not associated with clinically significant adverse events, the study design did not allow a definitive differentiation between sedation-related hypoventilation and other perioperative factors contributing to transient respiratory changes. Furthermore, intraoperative fluid administration differed significantly between groups, potentially reflecting hemodynamic management related to dexmedetomidine-associated vasodilation. This difference may have influenced perioperative hemodynamic and arterial blood gas parameters and should therefore be considered a potential confounding factor. Fourth, although the study was conducted in a randomized double-blind manner with standardized anesthetic protocols, formal assessment of blinding effectiveness was not performed. Although the study was designed as a double-blind trial using identical infusion protocols, complete preservation of blinding may have been difficult because dexmedetomidine can produce clinically recognizable sedative and hemodynamic effects. Therefore, the possibility of partial functional unblinding cannot be completely excluded. In addition, residual perioperative confounding factors that were not fully controlled may have influenced some secondary outcomes. Fifth, postoperative outcomes were evaluated only during the first 48 hours after surgery. Therefore, the potential long-term effects of perioperative dexmedetomidine administration on functional recovery, pulmonary complications, chronic pain, and prolonged opioid consumption could not be assessed. Finally, this study was conducted at a single center in a relatively selective patient population undergoing VATS lobectomy with combined epidural and general anesthesia. Therefore, the findings may not be generalizable to institutions using different perioperative anesthetic or analgesic protocols or to broader thoracic surgical populations. In addition, inclusion of patients with ASA physical status III may have introduced heterogeneity in perioperative risk profiles, which should be considered when interpreting the generalizability of the findings.

The implementation of perioperative dexmedetomidine protocols in thoracic surgery requires the consideration of institutional resources, staff familiarity, and monitoring capabilities. The dosing regimen used in this study (1 μg/kg loading followed by 0.5 μg/kg/h maintenance) provided clinically meaningful analgesic benefits while maintaining an acceptable safety profile. Institutions considering the incorporation of dexmedetomidine into thoracic anesthesia protocols should also assess its cost-effectiveness, as reduced opioid consumption and improved recovery may offset higher medication costs.

## Conclusions

This prospective randomized study demonstrates that perioperative dexmedetomidine infusion reduces cumulative postoperative opioid requirements and improves patient satisfaction after VATS lobectomy without increasing adverse events. Dexmedetomidine was also associated with lower pain scores at 1 hour postoperatively, although this difference was not sustained throughout the postoperative observation period.

## Funding

This work was supported by 2-year Research Grant from Pusan National University.

## Figures and Tables

**Table 1:** Basic demographic data

	Group D (n = 31)	Group S (n = 30)	p-value
Sex (M/F)	19/12	19/11	0.869
Age (yrs)	65.61 (13.2)	64.13 (10.4)	0.652
Height (cm)	161.22 (7.01)	161.15 (8.79)	0.902
Weight (kg)	61.67 (8.38)	63.86 (7.19)	0.375
BMI (kg/m^2^)	23.66 (2.39)	24.68 (2.87)	0.35
HTN	6 (19.4)	10 (33.3)	0.255
DM	3 (9.7)	7 (23.2)	0.15
Smoking	9 (29)	8 (26.7)	0.837
ASA physical status (I/II/III)	2/24/5	0/27/3	0.358

Basic information includes patient sex, age, height, weight, underlying conditions such as hypertension and diabetes mellitus, smoking history, and ASA physical status classification. There are no significant differences between the groups. Data are presented as mean (standard deviation) or number (percentage). Group D, dexmedetomidine group; Group S, saline group; BMI, body mass index; HTN, hypertension; DM, diabetes mellitus; ASA, American Society of Anesthesiologists. * p < 0.05, compared to the other group.

**Table 2:** Intraoperative data and postoperative variables

		Group D (n = 31)	Group S (n = 30)	p-value
Anesthesia time (min)		268 (78.37)	289.5 (69.93)	0.174
Operation time (min)		205.16 (78.87)	213.5 (61.77)	0.448
OLV time (min)		167.1 (77.82)	178.83 (60.31)	0.295
Rocuronium injection (count)		3.77 (1.67)	5.83 (2.78)	0.002*
Ephedrine injection (count)		1.87 (1.23)	1.47 (1.43)	0.281
Total Fluid administered (ml)		1538.71 (671.03)	1242.33 (850.7)	0.03*
EBL (ml)		169.35 (91.9)	176.67 (129.81)	0.668
Urine output (ml)		459.84 (411.24)	434.83 (404.58)	0.988
Intraoperative PCA dose (ml)		0.49 (1.12)	5.3 (5.27)	<0.001*
Bolus analgesics in PACU		0.68 (0.48)	0.93 (0.25)	0.013*
RASS in PACU	-2	7 (22.6)	8 (26.7)	0.046*
	-1	9 (29)	2 (6.7)	
	0	14 (45.2)	20 (66.7)	
	1	1 (3.2)	0	
Time to Aldrete score ≥ 9 (min)		35.32 (11.9)	39.17 (14.63)	0.383
ICU admission (count)		10 (32.3)	12 (40)	0.532
Admission duration (day)		6.35 (1.92)	7.6 (2.89)	0.091
Satisfaction		4.58 (0.62)	4.1 (0.66)	0.005*

Anesthesia time, operation time, one lung ventilation time, administered ephedrine count, EBL, urine output, time to Aldrete score ≥ 9, ICU admission, and admission duration do not differ between the two groups. The administered rocuronium count, bolus count of analgesics in the PACU, and administered patient-controlled analgesia dose are lower in Group D than in Group S. The amount of fluid administered is higher in Group D than in Group S. The RASS score in the PACU differs between the two groups. The satisfaction score of Group D is higher than that of Group S. Data are presented as mean (standard deviation) or count (percentage). Group D, dexmedetomidine group; Group S, saline group; OLV, one lung ventilation; EBL, estimated blood loss; PCA, patient-controlled analgesia; PACU, post-anesthesia care unit; RASS, Richmond Agitation-Sedation Scale; ICU, intensive care unit. * p < 0.05, compared to the other group.

**Table 3:** Adverse Events

Variable	Timepoint	Grade	Group D (n = 31)	Group S (n = 30)	p-value
Nausea	Post-op 1 hr	0	20 (64.5)	24 (80)	0.121
		1	7 (22.6)	1 (3.3)	
		2	4 (12.9)	5 (16.7)	
	Post-op 6 hr	0	22 (71)	23(76.7)	0.585
		1	8 (25.8)	7 (23.3)	
		2	(3.2)	0 (0)	
	Post-op 24 hr	0	27 (87.1)	26 (86.7)	0.96
		1	4 (12.9)	4 (13.3)	
	Post-op 48 hr	0	26 (83.9)	28 (93.3)	0.246
		1	5 (16.1)	2 (6.7)	
Vomiting			0	0	-
Bradycardia (HR < 60)	OLV 30 m		3	3	-
	OLV 1 hr		7 (22.6)	2 (6.7)	0.08
	Post-op 1 hr		4 (12.9)	0	0.044*
Hypotension (SBP < 90)	OLV 30 m		13 (41.9)	6 (20)	0.064
	OLV 1 hr		9 (29)	2 (6.7)	0.024*
Shivering			0	0	-
Respiratory depression			0	0	-

Nausea, vomiting, shivering, and respiratory depression do not differ between the two groups. The frequencies of bradycardia 1 hour after surgery and hypotension 1 hour after one-lung ventilation are higher in Group D. Data are shown as counts (percentages). Group D, dexmedetomidine group; Group S, saline group; HR, heart rate; SBP, systolic blood pressure; OLV, one-lung ventilation. * p < 0.05, compared to the other group.
